# Proteomic Insights into the Mechanism by Which Ferulic Acid Promotes Skeletal Muscle Fiber Type Conversion in Mongolian Horses

**DOI:** 10.3390/biology15060481

**Published:** 2026-03-18

**Authors:** Wendian Gong, Wenqi Ding, Tugeqin Bou, Lin Shi, Yanan Lin, Xiaoyuan Shi, Zheng Li, Huize Wu, Manglai Dugarjaviin, Dongyi Bai

**Affiliations:** Key Laboratory of Equus Germplasm Innovation (Co-Construction by Ministry and Province), Ministry of Agriculture and Rural Affairs, Inner Mongolia Key Laboratory of Equine Science Research and Technology Innovation, Equus Research Center, College of Animal Science, Inner Mongolia Agricultural University, Hohhot 010018, China; gongwendian1996@outlook.com (W.G.); dingwenqi0331@gmail.com (W.D.); tvgqin@gmail.com (T.B.); 19832607527@163.com (L.S.); linyanan@emails.imau.edu.cn (Y.L.); xiaoyuans2021@163.com (X.S.); lzheng0511@sina.com (Z.L.); whz020419@163.com (H.W.); dmanglai@163.com (M.D.)

**Keywords:** ferulic acid, skeletal muscle, proteomics, ferroptosis

## Abstract

Ferulic acid is a bioactive phenolic compound with potential benefits for skeletal muscle health. In this study, Mongolian horses were used as experimental subjects and were orally administered ferulic acid at doses of 5, 10, or 15 g per horse per day for 40 consecutive days. Muscle biopsy samples were analyzed using proteomics to assess fiber type composition and regulatory protein expression. Ferulic acid supplementation increased the proportion of fast-twitch fibers and upregulated key differentiation factors such as MUSTN1, while modulating glycolysis, ECM remodeling, and calcium signaling pathways. Although ferulic acid induced moderate oxidative stress, it did not trigger classical ferroptosis. Collectively, these findings demonstrate that ferulic acid promotes fast-twitch fiber transformation in horses through coordinated metabolic and signaling mechanisms, highlighting its potential as a nutritional strategy to enhance skeletal muscle adaptability and athletic performance in horses.

## Simple Summary

Ferulic acid (FA), a natural compound from traditional Chinese medicine, supports muscle health. Oral administration of ferulic acid in Mongolian horses can increase the proportion of fast-twitch muscle fibers, thereby enhancing speed and strength. Proteomic analysis showed FA promotes these fibers by regulating energy metabolism, muscle structure, and calcium signaling without inducing harmful cell damage.

## 1. Introduction

Skeletal muscle is one of the largest organs in animals, not only supporting motor function but also playing a crucial role in energy metabolism regulation and endocrine modulation [1]. Based on differences in oxidative capacity, myoglobin content, and mitochondrial abundance, skeletal muscle fibers are generally classified into slow-twitch and fast-twitch types [2]. Currently, classification based on myosin heavy chain (MyHC) isoforms is widely accepted, including slow oxidative fibers (Type I, MyHC I), fast oxidative fibers (Type IIA, MyHC IIA), intermediate fibers (Type IIX, MyHC IIX), and fast glycolytic fibers (Type IIB, MyHC IIB) [3]. These fiber types exhibit distinct physiological and metabolic characteristics, and their composition is closely linked to muscle performance, exercise capacity, atrophy, and metabolic disorders [4]. Equine skeletal muscle demonstrates unique adaptive features. Studies have shown that the maximum shortening velocity of fast-twitch (Type II) fibers can be up to 10 times greater than that of slow-twitch (Type I) fibers in adult horses, a difference that far exceeds that observed in mice and rats (approximately 3–4 fold) [5]. At the genetic level, muscle fiber type composition in horses is primarily regulated by MYH1, MYH2, and MYH7, which encode MyHC IIX/D, IIA, and I, respectively. Notably, MYH4, which encodes the IIB isoform, has lost its function during equine evolution, distinguishing horses from many other mammals [5]. In Mongolian horses, locomotor performance is closely associated with muscle fiber composition: a higher proportion of fast-twitch fibers enhances sprinting speed, while an increased proportion of slow-twitch fibers improves endurance [6]. Thus, the inherent remodeling of muscle fiber types provides a theoretical basis for improving equine athletic performance through nutritional interventions. Beyond their performance traits, Mongolian horses exhibit distinctive skeletal muscle metabolic characteristics shaped by long-term adaptation to harsh environments, including cold climates, limited nutrition, and sustained locomotion [7]. These conditions have favored a muscle phenotype with high oxidative capacity, flexible energy substrate utilization, and pronounced fiber-type plasticity [8]. Such metabolic and structural features render Mongolian horse skeletal muscle highly responsive to nutritional and signaling cues that regulate fiber-type remodeling, making this breed a biologically relevant model for investigating diet-mediated modulation of fast-twitch muscle development.

Ferulic acid (FA) is a natural phenolic compound widely found in traditional Chinese medicine, such as Angelica, Ligusticum chuanxiong, Cimicifuga, and rhizoma spargani [9]. It has low toxicity and exhibits multiple biological activities, including anti-inflammatory, antioxidant, antibacterial, anticancer, and antidiabetic effects [10]. Owing to these properties, FA has garnered increasing research attention for its potential role in regulating skeletal muscle fiber-type transformation across species. Studies have reported that FA increases the proportion of fast-twitch fibers in zebrafish [11], while promoting slow-twitch fiber formation in pigs [12] and sheep [13]. In addition, FA, as a highly bioactive phenolic compound, also possesses significant redox-regulating properties. Accumulating evidence indicates that FA can modulate intracellular reactive oxygen species (ROS), lipid peroxidation, and iron homeostasis—key determinants of ferroptosis, a regulated form of cell death driven by iron-dependent lipid peroxidation. Ferroptosis has recently been implicated in skeletal muscle homeostasis, regeneration, and pathological remodeling, suggesting that redox- and metabolism-targeting compounds such as FA may influence muscle phenotypes partly through ferroptosis-related mechanisms [14]. However, due to significant interspecies differences in muscle regulation, the effect of FA on equine skeletal muscle remains uncharacterized.

Our previous research on muscle satellite cells in Mongolian horses revealed that FA may promote fast-twitch muscle gene expression while suppressing slow-twitch gene expression by activating the PDK1/HIF signaling pathway. Nonetheless, its in vivo effects and specific regulatory mechanisms in horses are still unclear [15]. Considering both the species-specific responses to FA and the distinct genomic features of Mongolian horses (e.g., the evolutionary inactivation of MYH4), it is essential to elucidate the molecular networks and proteomic landscape underlying FA-induced muscle fiber transformation in vivo. Given accumulating evidence that ferulic acid (FA) promotes the expression of fast-twitch-fiber-associated genes and modulates skeletal muscle metabolic characteristics, we formulated the following hypotheses: FA supplementation would promote a shift in skeletal muscle toward a fast-twitch-dominant phenotype; this transition would be accompanied by coordinated proteomic remodeling favoring glycolytic metabolism and fast-twitch-associated contractile proteins; and FA-induced metabolic reprogramming would be associated with modulation of ferroptosis-related signaling pathways in muscle tissue.

Based on these hypotheses, the present study aimed to systematically evaluate the effects of FA supplementation on skeletal muscle fiber-type composition and global proteomic remodeling in Mongolian horses. Specifically, we sought to determine whether FA induces a shift toward fast-twitch fibers using immunofluorescence and histological analyses and to characterize the associated changes in protein expression profiles and metabolic pathways using quantitative proteomics. In addition, given the strong redox bioactivity of FA, we further investigated whether FA-induced muscle remodeling is accompanied by alterations in ferroptosis-related pathways.

## 2. Materials and Methods

### 2.1. Sample Collection

Thirteen healthy male Mongolian horses (Ujumqin white horses) of the same age and with consistent genetic backgrounds were selected and randomly assigned to four groups, including three control horses and three horses in each FA-treated group. According to stud farm pedigree records, the ages of horses in each experimental group were comparable: the control group was 72 ± 3.2 months old, the 5 g group was 72 ± 2.35 months old, the 10 g group was 72 ± 2.9 months old, and the 15 g group was 72 ± 3.0 months old. The control group (NC) did not receive ferulic acid (FA) and was administered an equivalent volume of water daily. The treatment groups received different doses of FA: the low-dose group received 5 g/horse/day, the medium-dose group received 10 g/horse/day, and the high-dose group received 15 g/horse/day.

All animals were maintained under standardized pasture conditions and identical management practices throughout the study period. To ensure consistent nutritional intake, all horses were provided with the same quantity of basal forage. Each day, the designated dose of ferulic acid powder (Shaanxi Sain Biotechnology Co., Ltd., Xi’an, China) was accurately weighed, prepared as a suspension in water, thoroughly mixed, and administered via oral gavage using a sterilized veterinary gavage tube. Horses in the control group received the same volume of water via the same method. The experimental period lasted 40 days. After the completion of the gavage experiment, samples were collected by a professional veterinarian under sterile conditions following standardized procedures and intravenous anesthesia. Muscle tissue was obtained from the gluteus medius of each horse at a depth of approximately 3 cm, with a size of about 1 cm^3^. Collected samples were immediately rinsed with phosphate-buffered saline (PBS). Some samples were fixed in formaldehyde solution for subsequent histological analysis, while the remaining samples were rapidly frozen in liquid nitrogen and stored at −80 °C for molecular biological studies.

### 2.2. Immunofluorescence

Fixed tissues or cells were permeabilized with 0.3% Triton X-100 for 15 min, followed by three washes with PBS. The samples were then blocked with 5% Bovine Serum Albumin (BSA) for 1 h. Primary antibodies against myosin heavy chain (MyHC) isoforms, including MyHC I (slow-twitch muscle fibers) and MyHC IIA (fast-twitch muscle fibers), were incubated at 4 °C for 15–16 h, followed by incubation with corresponding fluorescent secondary antibodies for 2 h. Finally, the samples were stained with 4′,6-diamidino-2-phenylindole (DAPI) at room temperature for 15 min and observed under a fluorescence microscope for imaging. The antibody information for utilized in immunofluorescence assay is provided in Table 1.

### 2.3. Protein Extraction and Digestion

Tissue samples were retrieved from the −80 °C freezer and ground into a fine powder under cryogenic conditions. The powdered tissue was immediately transferred into pre-chilled centrifuge tubes containing liquid nitrogen. An appropriate volume of SDT buffer (4% sodium dodecyl sulfate, 100 mM dithiothreitol, 100 mM Tris-HCl, pH 7.6) and 1% Dithiothreitol (DTT) (*v*/*v*) was added for protein solubilization. The mixture was thoroughly vortexed and subjected to ultrasonication in an ice-water bath at 4 °C for 5 min to ensure complete cell lysis. Subsequently, the lysates were incubated at 95 °C for 8–15 min, cooled at 4 °C for 2 min, and centrifuged at 12,000× *g* for 15 min at 4 °C. The supernatant was collected, supplemented with sufficient iodoacetamide (IAM), and incubated in the dark for 1 h at room temperature for alkylation.

To precipitate the proteins, four volumes of pre-chilled acetone (−20 °C) were added, and the mixture was incubated at −20 °C for at least 2 h. Samples were centrifuged again at 12,000× *g* for 15 min at 4 °C. The resulting pellet was washed with 1 mL of cold acetone, centrifuged, and air-dried to obtain the total protein fraction. The protein pellet was then dissolved in an appropriate volume of Dissolution Buffer (DB).

For protein enrichment, Bio-Rad ProteoMiner™ (Hercules, CA, USA) beads were added to a 1.5 mL centrifuge tube and mixed with protein extracted from muscle tissue lysate, followed by vertical rotation at room temperature for 2 h. After incubation, the mixture was centrifuged at 10,000× *g* for 5 min at 4 °C, and the supernatant was discarded. The beads were washed repeatedly with Wash Buffer (Thermo Scientific, Waltham, MA, USA). For elution, 0.4 mL of 1% trifluoroacetic acid (TFA) was added to the beads, mixed by inversion for 10 min, and the supernatant was collected. This elution step was repeated twice. The combined supernatants were freeze-dried to obtain protein powder.

The dried protein was resuspended in a denaturation buffer [8 M urea, 100 mM Triethylammonium bicarbonate buffer (TEAB), pH 8.5]. For reduction, 1 M DTT was added, and the sample was incubated at 56 °C for 1 h. After cooling at 4 °C for 2 min, IAM was added, and the sample was incubated in the dark at room temperature for 1 h. After centrifugation (12,000× *g*, 15 min, 4 °C), the supernatant was collected. Proteins were precipitated by adding four volumes of cold acetone and incubating at −20 °C for at least 4 h. The final pellet was collected by centrifugation, air-dried, and redissolved in protein solution [8 M urea, 100 mM Triethylammonium bicarbonate (TEAB), pH 8.5] for further analysis.

### 2.4. Protein Quantification and Enzymatic Digestion

Protein concentrations were determined using the Bradford assay (Leagene, Beijing, China). A bovine serum albumin (BSA) standard solution was prepared according to the kit instructions, with a concentration gradient ranging from 0 to 0.5 µg/µL. Various concentrations of BSA standards and serial dilutions of the test samples were added to a 96-well microplate (20 µL per well), with each condition performed in triplicate. Subsequently, 180 µL of Coomassie Brilliant Blue G-250 staining solution was added to each well, and the plate was incubated at room temperature for 5 min. Absorbance was measured at 595 nm using a microplate reader (Thermo Fisher Scientific, Waltham, MA, USA). A standard curve was generated from the BSA standards, and the protein concentrations of the test samples were calculated accordingly.

To assess protein quality, 20 µg of each sample was subjected to Sodium dodecyl sulfate polyacrylamide gel electrophoresis (SDS-PAGE) using a 12% polyacrylamide gel. Electrophoresis was performed at 80 V for 20 min (stacking gel) and 120 V for 90 min (separating gel). The gel was stained with Coomassie Brilliant Blue R-250 (Bio-Rad, Hercules, CA, USA) and destained until protein bands were clearly visible.

For enzymatic digestion, protein samples were dissolved in DB buffer (8 M urea, 100 mM TEAB, pH 8.5) to a final volume of 100 µL. Trypsin and 100 mM TEAB were added, and the samples were incubated at 37 °C for 4 h. A second aliquot of trypsin and CaCl_2_ was then added for overnight digestion at 37 °C. After digestion, formic acid was added to adjust the pH to <3, followed by centrifugation at 12,000× *g* for 5 min at room temperature. The supernatant was collected and passed slowly through a C18 desalting column.

The column was washed three times with a wash solution containing 0.1% formic acid and 3% acetonitrile. Peptides were then eluted using an elution buffer consisting of 0.1% formic acid and 70% acetonitrile. The eluate was collected and freeze-dried for subsequent analysis.

### 2.5. DIA-Based LC-MS/MS Analysis

The mobile phases were prepared as follows: mobile phase A consisted of 100% water with 0.1% formic acid, and mobile phase B consisted of 80% acetonitrile with 0.1% formic acid. Freeze-dried peptide samples were reconstituted in 10 µL of mobile phase A, centrifuged at 14,000× *g* for 20 min at 4 °C, and the supernatant was collected. A total of 200 ng of peptide was loaded for analysis by liquid chromatography–tandem mass spectrometry (LC-MS/MS).

Chromatographic separation was performed on a Vanquish Neo Nano UHPLC system (Thermo Fisher Scientific, Waltham, MA, USA). A C18 trap column (5 mm × 300 µm, 5 µm, Thermo, Waltham, MA, USA Cat# 174500) was used in combination with a C18 analytical column (PepMap™ Neo UHPLC, 150 µm × 15 cm, 2 µm, Thermo, Waltham, MA, USA Cat# ES906), maintained at 50 °C in a column oven.

Mass spectrometry was performed on a Thermo Orbitrap Astral mass spectrometer equipped with an Easy-Spray™ electrospray ionization (ESI) source (Thermo, Waltham, MA, USA). The spray voltage was set to 1.9 kV, and the ion transfer tube temperature was maintained at 290 °C. Data were acquired in data-independent acquisition (DIA) mode. The full MS scan range was *m*/*z* 380–980, with a resolution of 240,000 at *m*/*z* 200. The automatic gain control (AGC) target was set to 500%, and the precursor isolation window was set to 2 Th. A total of 300 DIA windows were used.

The normalized collision energy (NCE) was set to 25%, and fragment ions were acquired over an m/z range of 150–2000. The Orbitrap Astral resolution (Thermo, Waltham, MA, USA) for MS/MS scans was set to 80,000, and the maximum injection time was 3 ms. Raw mass spectrometry data files were saved in .raw format for further analysis.

### 2.6. Mass Spectrometry (MS) Data Analysis

The raw data files were analyzed using DIA-NN software (v.1.8.1) with protein sequences obtained from the NCBI protein database. The search parameters were set as follows: precursor mass tolerance of 10 ppm and fragment mass tolerance of 0.02 Da. Carbamidomethylation of cysteine was set as a fixed modification, and up to two missed cleavages were allowed. To improve the reliability of the results, DIA-NN further filtered the search results, considering only peptide-spectrum matches (PSMs) with a confidence ≥ 99% as reliable and retaining high-confidence peptides and proteins accordingly. Subsequently, false discovery rate (FDR) validation was performed for both peptides and proteins, and those with an FDR greater than 1% were removed. After obtaining the stringently filtered peptides and proteins, fold change (FC) was used to define differentially expressed proteins (DEPs). All statistical analyses were conducted using R software (version 4.4.1).

### 2.7. Functional Analysis of Proteins and DEP

Use the Interproscan software (v.5.64-96.0) for GO and IPR functional annotation (including Pfam, PRINTS, ProDom, SMART, ProSite, PANTHER databases), and perform functional protein family and pathway analysis on the identified proteins using COG and KEGG [16]. Carried out volcano plot analysis, cluster heatmap analysis, as well as pathway enrichment analysis using GO, IPR and KEGG [14], and used the STRING DB software (v.12.5) to predict possible protein–protein interactions (http://STRING.embl.de/, accessed on 3 August 2024) [17].

### 2.8. Cultivation and Identification of MuSCs from Mongolian Horses

Skeletal muscle tissue from 2-year-old Mongolian horses was collected, rinsed in physiological saline with added antibiotics, and transported to the laboratory for further experiments. The skeletal muscle was subjected to enzymatic digestion to isolate primary muscle satellite cells. The cells were cultured in a growth medium consisting of 10% fetal bovine serum (FBS, Gibco, London, UK) and 90% DMEM (Gibco, London, UK) in a 37 °C incubator with 5% CO_2_. After passage and purification, when the cells reached approximately 60% confluency, the medium was switched to a differentiation medium containing 2% horse serum to induce myogenic differentiation. Different concentrations of FA were dissolved and added to the culture dishes for treatment.

### 2.9. CCK8 Assay

Cells were seeded into 96-well plates at an appropriate density, with seven 96-well plates in total. Different concentrations of FA (0 ng/mL, 100 ng/mL, 200 ng/mL, 300 ng/mL, 400 ng/mL, 500 ng/mL, and 600 ng/mL) were added, with three replicates for each concentration. At 24, 48, 72, 96, and 168 h of incubation, 10 μL of CCK8 reagent was added to each well, followed by a 4 h incubation. Absorbance was measured at 450 nm using a microplate reader.

### 2.10. Validation and Data Analysis by qRT-PCR

The total cellular RNA was extracted from the collected cells, and quantitative PCR (qPCR) was carried out in triplicate using the SYBR Green PCR Master Mix Kit (TaKaRa, Beijing, China) and the 7500 Real-Time PCR System (Applied Biosystems, Waltham, MA, USA). All steps were performed in accordance with the manufacturer’s protocols. The specific primers employed in the qPCR assays are detailed in Table 2. Primer information for qRT-PCR is shown in Table 2.

### 2.11. Quantification and Statistical Analysis

Quantitative analysis of immunofluorescence staining was performed using ImageJ software (v.1.54, National Institutes of Health, Bethesda, MD, USA). For each animal, at least five randomly selected microscopic fields were analyzed. The area of fast-twitch muscle fibers (e.g., MyHC-II–positive signal) was measured and normalized to the total muscle area to obtain the proportion of fast-twitch fibers. Centralized nuclei were quantified manually using ImageJ software by counting nuclei located within the sarcoplasm and not in contact with the sarcolemma. The number of centralized nuclei was normalized to the total number of muscle fibers in each microscopic field. The mean value from all fields of each animal was used as one biological replicate.

Effect sizes for group comparisons were quantified using eta squared (*η^2^*), representing the proportion of total variance explained by the grouping factor. For each *η^2^* estimate, 95% confidence intervals were calculated using a non-parametric bootstrap approach with 1000 resamples. All statistical analyses were performed in R (version 4.2.3) using the packages effect size and boot.

For quantitative real-time PCR analysis, relative gene expression levels were calculated using the 2^−ΔΔCt^ method with GAPDH as the internal reference gene.

All statistical analyses were performed using SPSS software (IBM, v.1.54m Armonk, NY, USA). Each data point represents one individual animal (control, *n* = 4; FA-treated groups, *n* = 3 per group). Data are presented as mean ± standard deviation (SD). Differences among multiple groups were analyzed using one-way analysis of variance (ANOVA) followed by Tukey’s post hoc test. A value of *p* < 0.05 was considered statistically significant, and *p* < 0.01 was considered highly significant.

## 3. Results

### 3.1. Moderate Doses of Ferulic Acid Promote the Conversion of Skeletal Muscle Fibers to Fast-Twitch Type in Mongolian Horses

**Figure 1:**
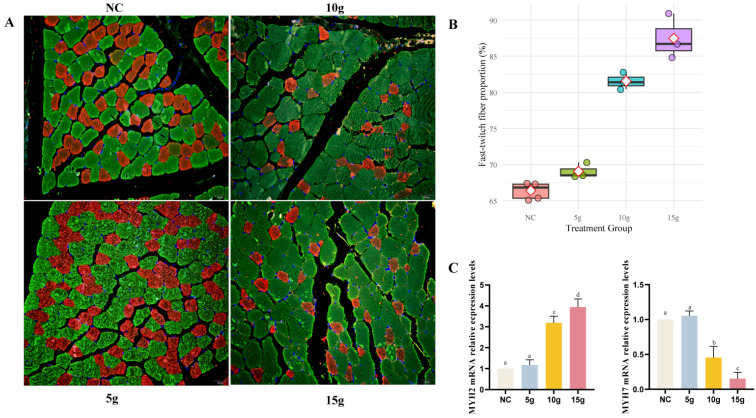
Ferulic acid (FA) supplementation increases the proportion of fast-twitch muscle fibers in Mongolian horses. (**A**) Representative immunofluorescence images of gluteus muscle sections from Mongolian horses in the control (no FA), 5 g FA, 10 g FA, and 15 g FA groups. Slow-twitch and fast-twitch muscle fibers were identified by immunofluorescent staining and are shown in red and green, respectively. Scale bars = 50 μm. (**B**) Quantification of fast-twitch muscle fiber proportion in each group based on immunofluorescence analysis. The red diamond indicates the mean value. (**C**) Relative mRNA expression levels of MYH2 and MYH7 in the gluteus muscle of Mongolian horses from the control, 5 g FA, 10 g FA, and 15 g FA groups. Data are presented as mean ± SD. Different lowercase letters above the bars indicate significant differences among groups (*p* < 0.05), whereas bars sharing the same letter are not significantly different. Each data point represents one animal, and data are presented as mean ± SD (control, *n* = 4; FA-treated groups, *n* = 3 per group).

Immunofluorescence analysis showed that the proportion of fast-twitch muscle fibers in the gluteus medius of FA-treated Mongolian horses was significantly higher than that in the control group (Figure 1A). In the control group, fast-twitch fibers accounted for 67.72%, and daily supplementation with 5 g FA did not result in a significant change in muscle fiber composition, with fast-twitch fibers accounting for 71.22%. In contrast, the groups receiving 10 g and 15 g FA per day showed a significant increase in fast-twitch fiber proportion, reaching 80.43% and 84.83%, respectively (*p* < 0.001; Figure 1B), and these results were further validated by the mRNA expression levels of the marker genes MYH2 and MYH7, which showed consistent trends (Figure 1C). The proportion of centrally nucleated fibers (CNFs) in skeletal muscle was low in all groups, ranging from 0% to 4%, and no significant differences were observed between the FA-treated groups and the control group (*p* > 0.05). (Supplementary Figure S1) One-way analysis of variance revealed a highly significant between-group difference (F ([3,10]) = 121.5, *p* < 0.001), with a very large effect size (η^2^ = 0.97, 95% CI [0.93, 1.00]), indicating that the treatment groups accounted for approximately 97% of the variance in the outcome variable, thereby enhancing the robustness of the results.

### 3.2. Preliminary Proteomic Analysis of FA-Induced Muscle Fiber Type Transformation

**Figure 2:**
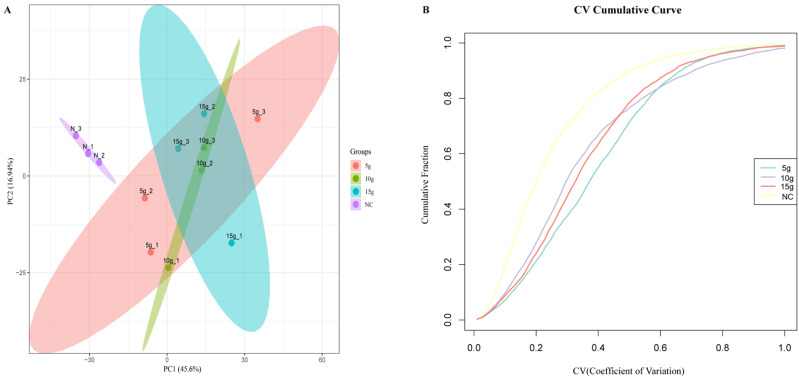
Multidimensional analysis and quality assessment of the proteomic data. (**A**) Principal component analysis (PCA) of proteomic profiles from gluteus muscle samples of Mongolian horses in the control (NC), 5 g FA, 10 g FA, and 15 g FA groups. The percentages on the axes indicate the variance explained by the first two principal components. (**B**) Distribution of coefficients of variation (CVs) of quantified proteins within each experimental group. CV analysis was used to evaluate the reproducibility and stability of proteomic measurements across biological replicates.

To investigate the molecular mechanisms by which FA promotes skeletal muscle fiber-type transformation in Mongolian horses, proteomic analysis was performed on gluteal muscle tissues from the NC and FA-treated groups (5 g, 10 g, and 15 g). Principal component analysis (PCA) showed that PC1 and PC2 accounted for 45.6% and 16.94% of the total variance, respectively. The NC samples were clearly separated from the FA-treated samples in the principal component space, whereas partial overlap was observed among the different FA dosage groups, indicating that FA primarily induces a global alteration in the skeletal muscle proteome rather than producing distinct dose-dependent clustering effects (Figure 2A). In addition, the NC samples clustered tightly, while the FA-treated samples exhibited a more dispersed distribution.

Analysis of the cumulative distribution of the protein expression coefficient of variation (CV) revealed that the NC group had a higher proportion of proteins with low CV values, indicating greater expression stability. In contrast, the CV distribution curves of the FA-treated groups were moderately right-shifted, suggesting increased variability in protein expression following FA treatment; however, intra-group consistency remained acceptable (Figure 2B). Collectively, these results demonstrate that FA intervention significantly remodels the skeletal muscle proteomic profile of Mongolian horses and that the proteomic data generated in this study exhibit good stability and reproducibility, providing a reliable foundation for subsequent differential protein identification and functional enrichment analyses.

### 3.3. Differential Protein Expression Induced by Varying Doses of Ferulic Acid in Mongolian Horse Skeletal Muscle

To evaluate the effects of different FA dosages on skeletal muscle protein expression in Mongolian horses, intergroup comparisons were performed using a two-sample *t*-test (*p* < 0.05). Compared with the NC group, a total of 932 (27 upregulated and 905 downregulated), 1084 (43 upregulated and 1041 downregulated), and 1273 (68 upregulated and 1205 downregulated) differentially expressed proteins (DEPs) were identified in the 5 g/d, 10 g/d, and 15 g/d FA-treated groups, respectively.

Notably, several proteins related to muscle fiber-type characteristics and metabolic remodeling showed dose-dependent alterations following FA supplementation. The intermediate-fiber marker MYH1 was significantly downregulated in both the 5 g and 10 g FA-treated groups compared with the NC group. In contrast, the fast-twitch muscle markers TNNI2 and TNNT3 were upregulated after FA treatment, with TNNI2 showing a significant increase in the 5 g group and TNNT3 being significantly elevated in the 5 g and 15 g groups relative to the NC group. In addition, the glycolytic enzymes PGK1 and LDHA were significantly increased in the 15 g group, whereas the mitochondrial fusion-related protein MFN1 was significantly decreased after FA supplementation. These changes collectively suggest that FA treatment promotes a shift in skeletal muscle toward a faster and more glycolytic phenotype, accompanied by suppression of mitochondrial oxidative characteristics.

**Figure 3:**
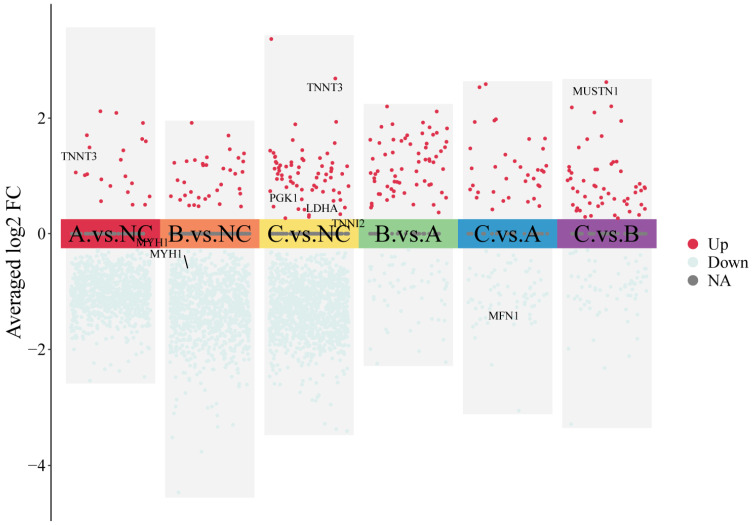
Grouped scatter plot analysis of different proteins in control group and FA group.

Pairwise comparisons among FA-treated groups identified 146, 130, and 164 DEPs in FA (5 g) vs. FA (10 g), FA (5 g) vs. FA (15 g), and FA (10 g) vs. FA (15 g), respectively (Figure 3). Overall, both the number of DEPs and the magnitude of expression changes increased with increasing FA dosage. Notably, several proteins associated with skeletal muscle differentiation and regeneration, such as MUSTN1 and MYOZ2, were significantly upregulated primarily under high-dose FA treatment rather than showing consistent changes across all FA-treated groups, suggesting that these proteins may be involved in later stages of FA-induced skeletal muscle remodeling.

### 3.4. GO Enrichment Analysis of Upregulated Proteins Provides Functional Insights into Skeletal Muscle Fiber Type Transformation

**Figure 4:**
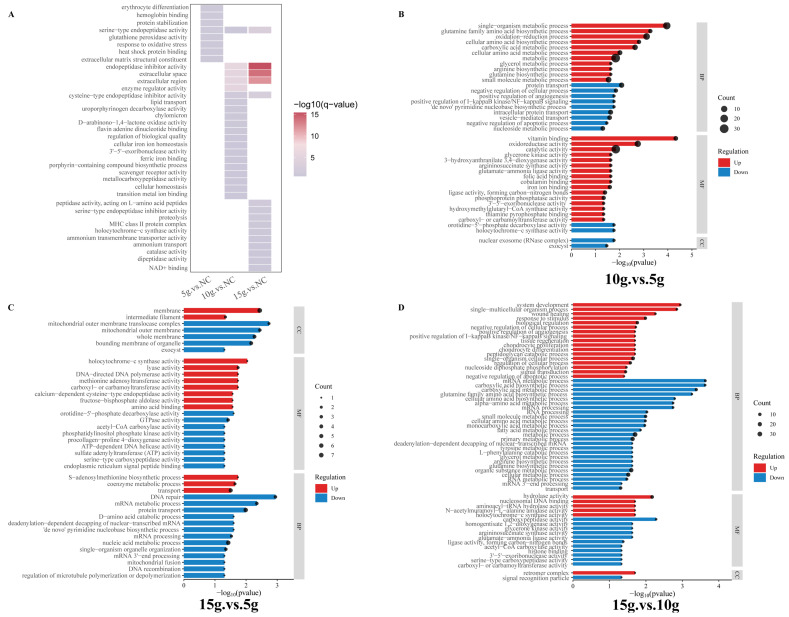
Gene Ontology (GO) enrichment analysis of upregulated proteins following ferulic acid (FA) supplementation. (**A**) GO enrichment analysis of proteins significantly upregulated in the 5 g, 10 g, and 15 g FA groups compared with the control (no FA) group. (**B**–**D**) Comparative GO enrichment analyses of differentially upregulated proteins between FA dose groups: FA (10 g) vs. FA (5 g), FA (15 g) vs. FA (5 g), and FA (15 g) vs. FA (10 g).

GO enrichment analysis was performed to identify biological processes significantly enriched following FA supplementation. Compared with the NC, both the 10 g and 15 g FA-treated groups were significantly enriched in processes related to extracellular matrix organization, although the specific enriched GO terms differed between the two dosage groups (Figure 4A). In the 5 g FA-treated group, upregulated proteins were mainly enriched in oxygen transport-related processes, such as erythrocyte differentiation and hemoglobin complex stabilization. The 10 g FA-treated group showed significant enrichment in processes associated with iron ion binding and intracellular iron ion homeostasis, whereas the 15 g FA-treated group was predominantly enriched in energy metabolism-related terms, including NAD^+^ binding (Figure 4A).

Inter-dose comparisons revealed that, relative to the 5 g group, upregulated proteins in the 10 g group were enriched in processes related to vitamin binding, amino acid metabolism, and iron ion binding (Figure 4B). In contrast, upregulated proteins in the 15 g group were enriched in calcium-dependent proteolysis, transamination reactions, and methionine metabolism-related processes (Figure 4C). In addition, comparison between the 10 g and 15 g groups showed that upregulated proteins in the 15 g group were significantly enriched in biological processes associated with tissue regeneration and system development (Figure 4D), despite no significant differences in muscle fiber-type proportions being observed between these two groups.

In the comparisons of NC vs. FA (5 g), NC vs. FA (10 g), and NC vs. FA (15 g), 88, 93, and 122 significantly downregulated GO terms were identified, respectively. Across all three comparisons, the downregulated differentially expressed proteins were predominantly enriched in translation- and ribosome-associated processes, RNA metabolism, and proteasome-mediated protein degradation, encompassing functional categories such as translation-related processes, gene expression, and protein metabolic process (Supplementary File S1).

In the comparisons of FA (10 g) vs. FA (5 g), FA (15 g) vs. FA (5 g), and FA (15 g) vs. FA (10 g), 86, 117, and 129 downregulated pathways were enriched, respectively. Among these, the downregulated GO pathways in FA (10 g) vs. FA (5 g) mainly involved processes such as cytoskeleton remodeling, regulation of actin–myosin complexes, vesicle-mediated protein transport, as well as mitochondrial function and energy metabolism (Supplementary File S2). FA (15 g) vs. FA (5 g) primarily involved cytoskeleton organization and myofiber structural remodeling, mRNA processing and post-transcriptional regulation, lipid metabolism, and vesicle-mediated protein transport (Supplementary File S3). FA (15 g) vs. FA (10 g) mainly involved mRNA processing and post-transcriptional regulation, amino acid and fatty acid metabolism, glycolysis, and proteasome-mediated protein degradation (Supplementary File S4). These downregulated pathways collectively indicate that, as the FA dose increases, there may be suppression of cytoskeletal and myofibrillar remodeling, dynamic changes in contractile complexes, post-transcriptional regulation, and multiple energy and substance metabolism pathways, thereby shaping the molecular and metabolic milieu for myofiber phenotype transformation.

### 3.5. KEGG Enrichment Analysis Reveals Pathways Underlying FA-Induced Skeletal Muscle Fiber Type Transformation

**Figure 5:**
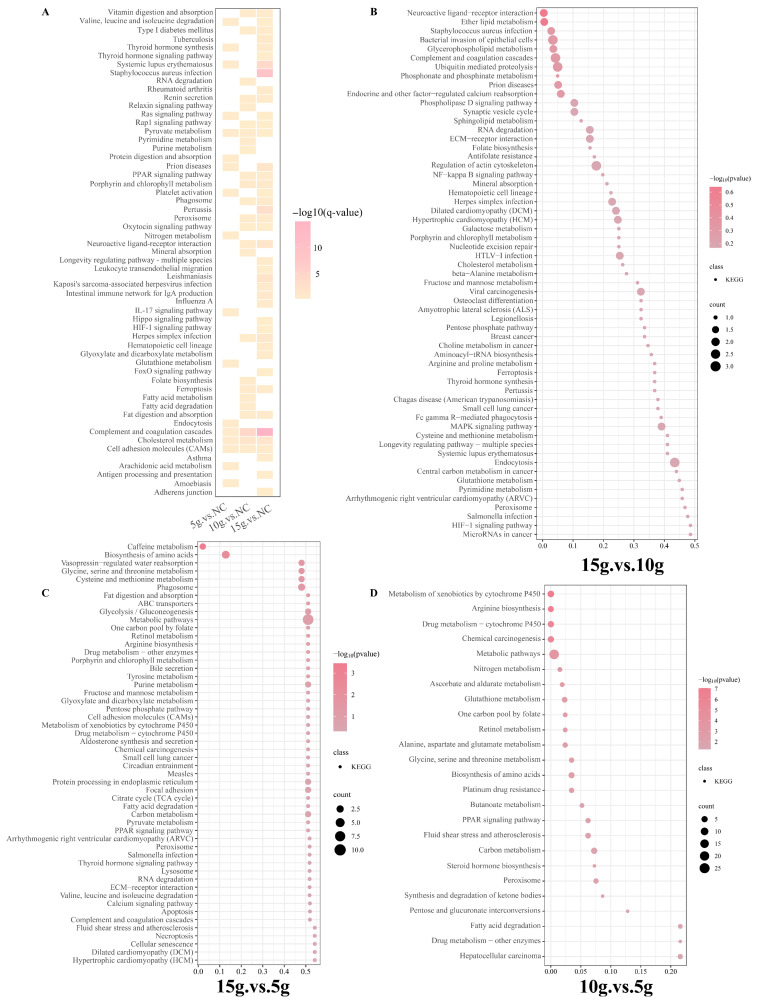
KEGG pathway enrichment analysis of upregulated proteins following ferulic acid (FA) supplementation. (**A**) KEGG pathway enrichment analysis of proteins significantly upregulated in the 5 g, 10 g, and 15 g FA groups compared with the control (no FA) group. (**B**–**D**) Comparative KEGG enrichment analyses of differentially upregulated proteins between FA dose groups: 10 g versus 5 g (**B**), 15 g versus 5 g (**C**), and 15 g versus 10 g (**D**).

To elucidate the potential pathways through which ferulic acid (FA) promotes skeletal muscle fiber-type transformation in Mongolian horses, KEGG pathway enrichment analysis was performed on the differentially expressed proteins identified across the comparison groups. Compared with the control group, FA-treated groups were significantly enriched in energy metabolism-related pathways, such as pyruvate metabolism, as well as the cell adhesion molecules (CAMs) pathway (Figure 5A) (Supplementary File S5). In addition, significant enrichment of ferroptosis- and iron metabolism-related pathways was also observed. Further inter-dose comparisons revealed that, in the comparison between the 15 g and 10 g groups, differentially expressed proteins were significantly enriched in multiple signaling pathways associated with skeletal muscle remodeling and cellular adaptive regulation, including the NF-κB, MAPK, HIF-1, and PPAR signaling pathways, as well as biosynthesis of amino acids, regulation of the actin cytoskeleton, and ECM–receptor interaction (Figure 5B) (Supplementary File S6). In the comparison between the 15 g and 5 g groups, differentially expressed proteins were predominantly enriched in core energy metabolism pathways, such as pyruvate metabolism, the tricarboxylic acid (TCA) cycle, and glycolysis/gluconeogenesis, accompanied by significant enrichment of the calcium signaling pathway, the renin–angiotensin system, and ECM–receptor interaction (Figure 5C) (Supplementary File S7). Furthermore, in the comparison between the 10 g and 5 g groups, differentially expressed proteins were significantly enriched in carbohydrate metabolism, fluid shear stress and atherosclerosis, and multiple other metabolism-related pathways, suggesting that FA exerts broad regulatory effects on the skeletal muscle metabolic network (Figure 5D) (Supplementary File S8).

### 3.6. IPR Domain Enrichment Analysis Suggests Multi-Mechanistic Coordination Underlies Fast-Twitch Muscle Fiber Transformation

To further elucidate the potential mechanisms through which FA regulates the transformation of skeletal muscle fiber types in Mongolian horses, we performed InterPro (IPR) domain enrichment analysis on the differentially expressed proteins identified in the control group and each FA treatment group (5 g, 10 g, and 15 g).

**Figure 6:**
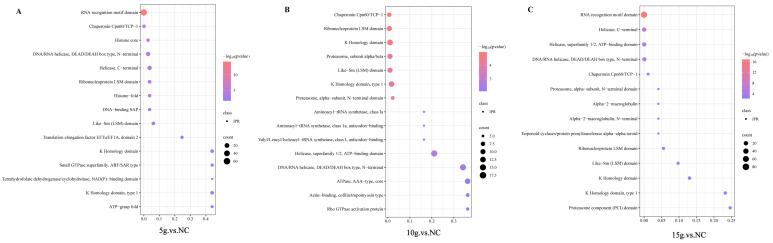
InterPro (IPR) enrichment analysis of upregulated proteins following ferulic acid (FA) supplementation. (**A**–**C**) IPR enrichment analysis of proteins significantly upregulated in the 5 g FA (**A**), 10 g FA (**B**), and 15 g FA (**C**) groups compared with the control (no FA) group.

The results revealed that all FA-treated groups were significantly enriched in conserved domains associated with RNA metabolism, including the “RNA recognition motif domain”, “Like-Sm (LSM) domain”, “K Homology domain”, and “K Homology domain, type 1”. These findings indicate widespread transcriptional and post-transcriptional regulation in skeletal muscle tissue. In parallel, domains associated with protein homeostasis, such as “Chaperonin Cpn60/TCP-1” and “DNA/RNA helicase, DEAD/DEAH box type, N-terminal”, were also significantly enriched, suggesting active mechanisms of protein folding, processing, and quality control in muscle cells. The consistent enrichment of these domains across all treatment groups suggests they may represent fundamental structural and functional components of skeletal muscle maintenance and basal metabolism, although these mechanisms alone are likely insufficient to drive fiber-type transformation toward the fast-twitch phenotype (Figure 6A–C).

More detailed comparisons revealed that the 5 g FA group lacked enrichment of several key co-regulatory domains observed in the 10 g and 15 g groups. Notably absent were domains such as “Alpha-2-macroglobulin, N-terminal” domain related to inflammation regulation, the “Proteasome component (PCI) domain” related to protein degradation, the “Aminoacyl-tRNA synthetase, class 1a, anticodon-binding” and several domains related to muscle structure and contraction, including “Actin-binding, cofilin/tropomyosin type”, “Rho GTPase activation protein”, and “Helicase, superfamily 1/2, ATP-binding domain” (Figure 6A–C).

The selective enrichment of these domains in the 10 g and 15 g groups suggests that higher doses of FA activate a broader and more integrated set of molecular pathways, including inflammatory response modulation, protein turnover, translational efficiency, and cytoskeletal remodeling. These pathways may collectively contribute to the observed shift toward fast-twitch muscle fiber characteristics.

Taken together, our findings suggest that FA does not promote muscle fiber-type transformation through a single molecular mechanism or pathway, but rather through the coordinated regulation of multiple functional domains. This multi-mechanistic model provides a more comprehensive explanation of how FA exerts its regulatory effects on skeletal muscle phenotypes in Mongolian horses.

### 3.7. Evaluation of the Potential Involvement of Ferroptosis in FA-Induced Fast-Twitch Muscle Fiber Transformation

Given the reported links between ferroptosis and muscle physiology, we investigated whether ferroptosis is involved in FA-induced muscle fiber-type transformation. Integrative analysis of proteomic and MuSC RNA-seq data revealed significant enrichment of ferroptosis-related pathways following FA treatment, suggesting a potential association with fast-twitch muscle development. To functionally assess this possibility, Mongolian horse MuSCs were treated with the ferroptosis inhibitor Ferrostatin-1 (Fer-1).

**Figure 7:**
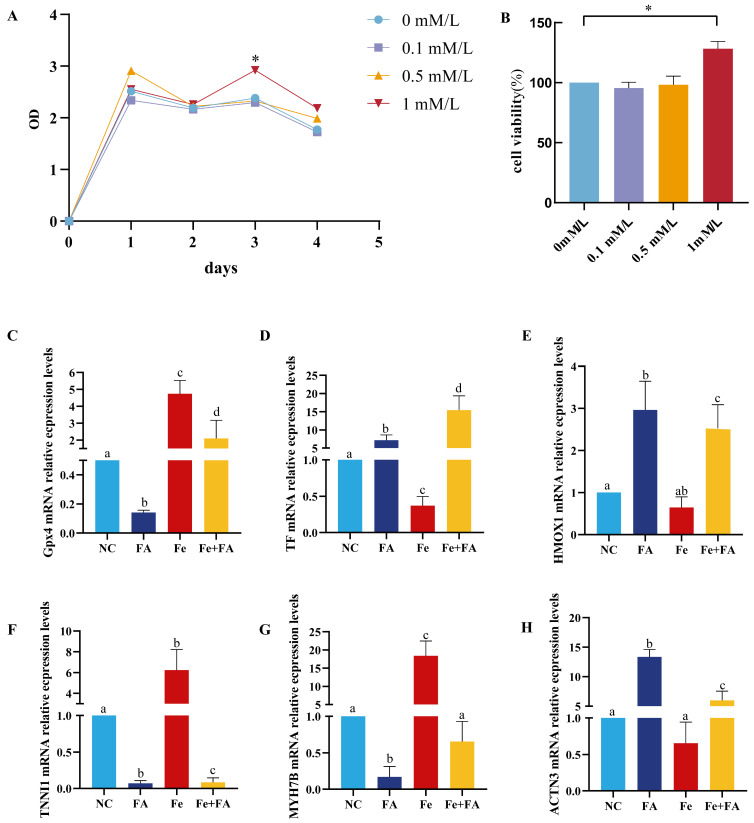
FA on ferroptosis-related pathways in Mongolian horse muscle satellite cells (MuSCs). (**A**) Cell proliferation assessed by CCK-8 assay in MuSCs treated with Ferrostatin-1, a ferroptosis inhibitor, in the presence or absence of FA. (**B**) Activity of MuSCs measured on day 3 of culture with Ferrostatin-1 and/or FA treatment. (**C**–**H**) mRNA expression levels of ferroptosis- and muscle fiber-related genes, including GPX4, TF, HMOX1, TNNI1, MYH7B, and ACTN3 (*n* = 3 biological replicates per group). Gene expression was quantified by qRT-PCR and normalized to an internal control. Data are presented as mean ± SD. Statistical significance was determined using one-way ANOVA followed by Tukey’s post hoc test. Different lowercase letters (a–d) above the bars indicate significant differences among groups at * *p* < 0.05; bars with the same letter indicate no significant difference.

CCK-8 assays showed that 1.0 mM Fer-1 significantly enhanced MuSC proliferation at day 3 compared with lower concentrations, and this dose was therefore used in subsequent experiments (Figure 7A,B). Four treatment groups were established: control, FA, Fer-1, and FA + Fer-1. In the FA group, GPX4 and TF expression remained unchanged, whereas HMOX1 was significantly upregulated, indicating increased oxidative stress without full activation of canonical ferroptosis (Figure 7C–E). Concurrently, FA significantly increased the fast-twitch marker ACTN3 and suppressed the slow-twitch markers TNNI1 and MYH7B (Figure 7F–H).

Fer-1 treatment alone significantly increased GPX4 expression while reducing TF and HMOX1 levels, confirming effective ferroptosis inhibition. Notably, Fer-1 also promoted slow-twitch fiber marker expression and suppressed ACTN3. In contrast, co-treatment with FA + Fer-1 maintained elevated ACTN3 expression and reduced TNNI1 and MYH7B levels despite effective ferroptosis inhibition. These results indicate that FA-induced fast-twitch muscle fiber transformation occurs independently of ferroptosis signaling, whereas ferroptosis inhibition alone favors slow-twitch muscle characteristics.

## 4. Discussion

FA, a major bioactive compound found in a variety of natural foods, is known for its notable medicinal and health-promoting properties [18]. Skeletal muscle fiber-type composition plays a pivotal role in muscle development, regeneration, athletic performance, and energy metabolism. However, the role of FA in regulating muscle fiber-type transformation in Mongolian horses and its underlying molecular mechanisms has remained largely unexplored. In this study, we combined phenotypic evaluation with proteomic profiling of the gluteus medius muscle from Mongolian horses supplemented with different doses of FA to systematically investigate its regulatory effects.

Ferroptosis is a recently identified form of programmed cell death characterized by iron-dependent lipid peroxidation and was formally named in 2012. However, related phenomena such as oxidative stress-induced cell death had been reported earlier [17]. Ferroptosis is now recognized as a widespread and evolutionarily conserved mode of cell death. In skeletal muscle, various cell death pathways—including apoptosis, necrosis, and autophagy—are involved in muscle degeneration and regeneration [14], with ferroptosis emerging as an important contributor. Fast-twitch fibers, due to their high oxidative metabolism, unique lipid profiles, and distinct iron-handling characteristics, may be particularly susceptible to ferroptosis. Therefore, an increase in fast-twitch fibers may be associated with activation of ferroptotic processes.

Our results demonstrated that daily supplementation with 10 g and 15 g FA significantly increased the proportion of fast-twitch muscle fibers, consistent with observations reported in zebrafish [11] but contrasting with findings in sheep and pigs, where FA was associated with a slow-twitch fiber shift [13,19]. These discrepancies likely reflect species-specific differences in muscle metabolism and regulatory networks, highlighting the complexity of FA-mediated muscle modulation across livestock species. Importantly, the proportion of centrally nucleated fibers remained consistently low across groups and showed no significant differences, indicating that FA-induced changes in fiber composition were unlikely to be driven by muscle fiber degeneration–regeneration processes.

Proteomic analysis provided mechanistic insight into these phenotypic changes. Among the differentially expressed proteins, MUSTN1 emerged as a key candidate due to its strong and dose-dependent upregulation in the 10 g and 15 g FA groups. MUSTN1 is predominantly expressed in Pax7-positive skeletal muscle satellite cells and plays a critical role in myogenic differentiation, fusion, and regeneration [20]. Recent studies further identify MUSTN1 as a potential target for genetic improvement in livestock and therapeutic intervention in muscle disorders [21]. Consistent with this interpretation, FA treatment also reduced the expression of the intermediate-fiber marker MYH1, increased the abundance of fast-twitch-associated proteins such as TNNI2 and TNNT3, enhanced glycolytic enzymes including PGK1 and LDHA, and decreased mitochondrial fusion-related proteins MFN1 and MFN2, collectively indicating a coordinated shift toward a faster and more glycolytic muscle phenotype. These findings suggest that FA may promote the differentiation of skeletal muscle satellite cells by upregulating MUSTN1, thereby facilitating the fiber-type transformation toward fast-twitch characteristics.

Functional enrichment analysis revealed that FA significantly upregulated pathways associated with the extracellular matrix (ECM), trace element absorption, lipid metabolism and glycolysis. The ECM plays a critical role in skeletal muscle growth and regeneration, with its mechanical properties and biochemical composition modulating satellite cell activation and renewal [22]. In parallel, enrichment of glycolytic and pyruvate metabolism pathways supports enhanced rapid energy production, a hallmark of fast-twitch muscle fibers [23]. The activation of lipid metabolism-related pathways, including PPAR signaling, is consistent with the established role of FA in regulating lipid utilization [24]. suggesting improved metabolic efficiency to meet fast-fiber energy demands.

The differences in protein expression profiles and pathway enrichment across increasing doses of FA suggested a multi-mechanistic and synergistic regulatory framework underlying its promotion of fast-twitch muscle fiber transformation, with higher FA doses showing a greater number of differentially expressed proteins and enriched pathways. InterPro (IPR) domain enrichment analysis further supported this notion, showing that while all treatment groups shared enrichment in domains related to RNA metabolism and protein homeostasis, the low-dose group (5 g) lacked several critical functional domains associated with inflammation suppression, protein degradation, and muscle contraction regulation, such as Alpha-2-macroglobulin [25] and PCI domain [26]. This suggests that the 5 g dose primarily supports basic cellular functions, whereas the 10 g and 15 g doses facilitate phenotypic conversion through more comprehensive molecular network regulation. Notably, the consistent enrichment of the ECM–receptor interaction pathway across multiple analyses (IPR, GO, and KEGG) suggests that FA may regulate muscle fiber type transformation by modulating the crosstalk between the extracellular matrix and muscle cells. This is in agreement with previous studies reporting that ECM-mediated signaling plays a vital role in communication between muscle stem cells and fast/slow muscle fibers, and in their self-renewal and differentiation processes [27]. Furthermore, the significant activation of the NF-κB and MAPK signaling pathways in the higher-dose groups indicates that FA may contribute to muscle fiber remodeling by balancing inflammatory responses and regenerative processes [28,29]. In addition, the enrichment of the HIF-1 signaling pathway suggests that FA might simulate a hypoxic environment, thereby enhancing glycolytic activity, which is consistent with the metabolic profile of fast-twitch muscle fibers. Taken together, these results indicate that FA-mediated muscle fiber-type transformation is not driven by a single molecular mechanism or signaling pathway, but instead arises from the integrated modulation of multiple functional processes.

The observed enrichment of ferroptosis-related pathways is more likely to reflect a cellular response to FA-induced oxidative stress rather than a direct regulatory role in muscle fiber-type transformation. Notably, although ferroptosis-related pathways were significantly enriched in the FA-treated groups and the oxidative stress marker HMOX1 was markedly upregulated, PCR validation demonstrated that the expression levels of classical core regulators of ferroptosis, such as GPX4 [30] and TF [31], did not exhibit significant changes. These findings indicate that FA did not substantially activate the canonical ferroptosis pathway. In addition, intervention experiments using the ferroptosis inhibitor Ferrostatin-1 revealed effects opposite to those of FA, characterized by the promotion of slow-twitch muscle fiber phenotypes. However, in the combined FA + Ferrostatin-1 group, expression of the fast-twitch muscle gene ACTN3 remained significantly upregulated [32], while slow-twitch markers TNNI1 [33] and MYH7B [34] continued to be suppressed. This provides further evidence that the FA-induced fast-twitch muscle transformation is independent of the ferroptosis pathway and may be mediated through alternative, ferroptosis-independent mechanisms. This highlights the importance of integrating omics-based pathway analysis with functional validation, as pathway enrichment alone does not necessarily indicate a causative role in phenotypic outcomes.

Skeletal muscle fiber composition is shaped by both genetic background and environmental inputs. Although this study focused on Mongolian horses, the molecular pathways influenced by FA are evolutionarily conserved across mammals, suggesting potential broader relevance beyond a single breed. Several limitations should be acknowledged. First, plasma and skeletal muscle concentrations of FA were not directly quantified, as the present work was designed as a functional nutritional intervention rather than a pharmacokinetic investigation. Second, although fast fiber enrichment was supported by immunofluorescence staining, RT-qPCR, and quantitative proteomics, additional validation, such as Western blot analysis of selected proteins and more detailed morphometric analysis of muscle fibers, would further strengthen the structural interpretation. Importantly, histological examination did not reveal typical features of active muscle regeneration, suggesting that the observed changes are more likely attributable to fiber-type remodeling rather than de novo fiber formation. Furthermore, while proteomic reanalysis indicated enrichment of glycolytic and fast fiber-associated metabolic pathways, direct measurements of mitochondrial function, metabolite levels (e.g., glucose, lactate, and ATP), and neuromuscular junction structure were beyond the scope of this tissue-level molecular study. Nevertheless, nutritional intervention alone may be insufficient to induce definitive functional adaptation without mechanical loading. The absence of direct measurements of muscle strength, glycogen content, and lipid accumulation, together with the relatively small sample size, represents an additional limitation of this study. Future investigations combining FA supplementation with controlled exercise regimens and comprehensive functional assessments will be essential to establish causal links between molecular remodeling and performance outcomes.

Skeletal muscle fiber composition is shaped by both genetic background and environmental inputs. Although this study focused on Mongolian horses, the regulatory pathways influenced by FA are highly conserved across mammals, suggesting broader applicability beyond a single breed. Nevertheless, nutritional intervention alone may be insufficient to induce definitive functional adaptation without mechanical loading. The absence of direct measurements of muscle strength, glycogen content, and lipid accumulation, together with the relatively small sample size, represents a limitation of this study. Future investigations combining FA supplementation with controlled exercise regimens and comprehensive functional assessments will be essential to establish causal links between molecular remodeling and performance outcomes.

Ferulic acid promotes fast-twitch muscle fiber transformation through coordinated regulation of satellite cell differentiation, metabolic reprogramming, ECM remodeling, and inflammatory–regenerative balance, rather than via ferroptosis activation. These findings expand current understanding of FA-mediated muscle regulation and provide a theoretical basis for its potential application as a nutritional strategy to enhance athletic performance in horses.

## 5. Conclusions

In summary, this study systematically demonstrated the in vivo effects of ferulic acid on skeletal muscle fiber-type remodeling in Mongolian horses. Ferulic acid promotes fast-twitch muscle fiber formation through multi-level regulation involving energy metabolism, extracellular matrix remodeling, and calcium signaling pathways, accompanied by the upregulation of key regulatory factors such as MUSTN1. Notably, FA-induced muscle fiber transformation was not associated with activation of the canonical ferroptosis pathway. These findings suggest that ferulic acid modulates skeletal muscle plasticity through ferroptosis-independent mechanisms and may serve as a promising nutritional strategy for enhancing equine athletic performance.

## Figures and Tables

**Table 1:** Information on antibodies used in immunofluorescence.

Antibody	Source	Dilution	Information
Fast-muscle	Mouse	1:500	ab51263 (Abcam, Shanghai, China)
Slow-muscle	Rabbit	1:300	ab234431 (Abcam, Shanghai, China)

**Table 2:** The sequences of the primers used for quantitative real-time PCR.

Genes	Primer Sequence (50–30) Annealing	Amplicon Size
GPX4	F: TGTGGAAGTGGATGAAGGCC R: CTCTATGACCAGGGGCTCCT	159 bp
ACTN3	F: AACTACAAGAGCAACATCGACC R: GTGTGCTTATTGTCAAACACGA	80 bp
MYH7B	F: GTCTTCTCAAGAGACGAACACT R: GTCGAACAGTTTCTGGTTCATG	163 bp
TF	F: CTGTCGTGTTTGTTGTGGATAG R: GCTTATTGCAGGCGATTAAGAA	115 bp
TNNT1	F: CGTAAAAAGCCTCTGAACATCG R: ATACAGCACGTTGATCTCGTAT	219 bp
HMOX1	F: GAAGAACTTTCAGAAAGGCCAG R: TTCTCCTTGTTGTCCTCGATTT	111 bp
GAPDH	F: CATCAATGGATTTGGACGCATC R: ACACCATGTATTCTGGGTCAAT	117 bp

## Data Availability

No new data were created or analyzed in this study. Data sharing is not applicable to this article.

## Supplementary Materials

The following supporting information can be downloaded at: https://www.mdpi.com/article/10.3390/biology15060481/s1, Supplementary Figure S1: Validation of fast- and slow-twitch muscle marker genes; Supplementary File S1: GO functional enrichment of FA vs. NC; Supplementary File S2: GO functional enrichment of FA (10 g) vs. FA (5 g); Supplementary File S3: GO functional enrichment of FA (15 g) vs. FA (5 g); Supplementary File S4: GO functional enrichment of FA (15 g) vs. FA (10 g); Supplementary File S5: KEGG functional enrichment of FA vs. NC; Supplementary File S6: KEGG functional enrichment of FA (15 g) vs. FA (10 g); Supplementary File S7: KEGG functional enrichment of FA (15 g) vs. FA (5 g); Supplementary File S8: KEGG functional enrichment of FA (10 g) vs. FA (5 g).
