# Supplementary figures and images for: Proteomic Insights into the Mechanism by Which Ferulic Acid Promotes Skeletal Muscle Fiber Type Conversion in Mongolian Horses
Source: Biology (Basel). 2026 Mar 18;15(6):481. doi: 10.3390/biology15060481 (PMC13024172; doi:10.3390/biology15060481)

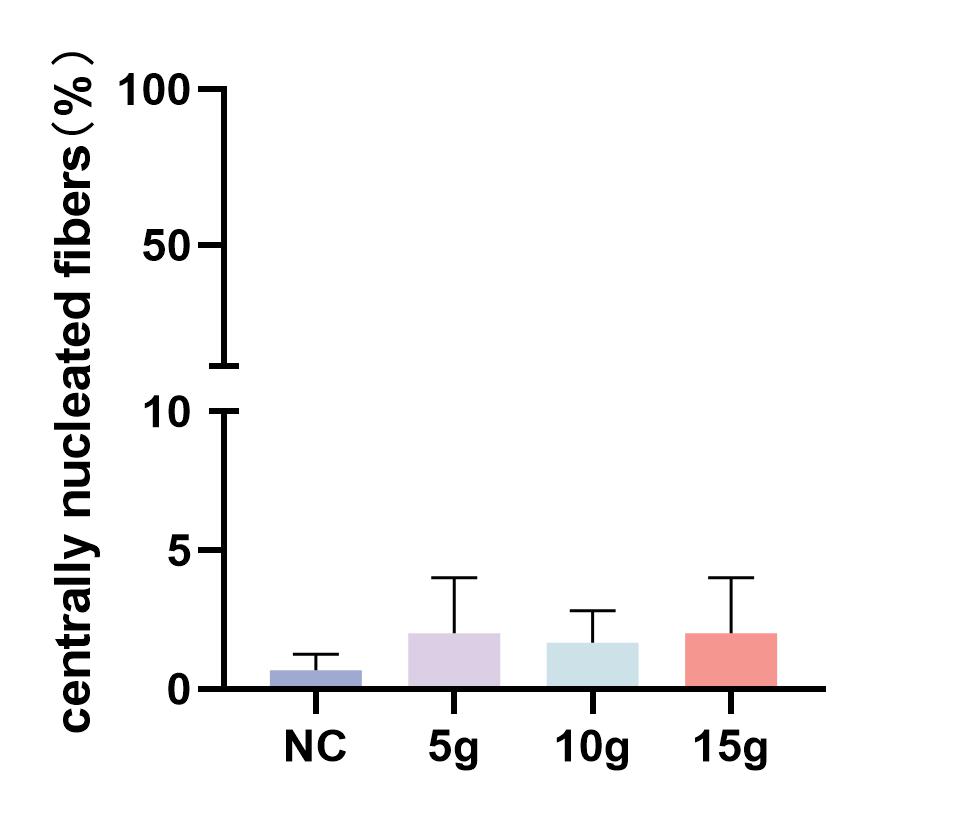

Supplement: Supplementary file 1 [file biology-15-00481-s001.zip › Supplementary Figure S1 centrally nucleated fibers(%).jpg]
